# Case report: A case of duodenal adenocarcinoma achieving significantly long survival treating with immune checkpoint inhibitors and chemotherapy without positive biomarkers

**DOI:** 10.3389/fimmu.2022.1046513

**Published:** 2022-12-02

**Authors:** Xian Chen, Rui Zhou, Yong Li, Xin Qu, Yan-chun Qu, Wen-zhu Li, Yong-song Ye, Li-rong Liu, Yan-juan Zhu, Hai-bo Zhang

**Affiliations:** ^1^ The Second Clinical Medical School of Guangzhou University of Chinese Medicine, Guangzhou, China; ^2^ Department of Oncology, The Second Affiliated Hospital of Guangzhou University of Chinese Medicine, Guangdong Provincial Hospital of Traditional Chinese Medicine, Guangzhou, China; ^3^ Department of Image, The Second Affiliated Hospital of Guangzhou University of Chinese Medicine, Guangdong Provincial Hospital of Traditional Chinese Medicine, Guangzhou, China; ^4^ Guangdong-Hong Kong-Macau Joint Lab on Chinese Medicine and Immune Disease Research, The Second Affiliated Hospital of Guangzhou University of Chinese Medicine, Guangzhou, China; ^5^ Guangdong Provincial Key Laboratory of Clinical Research on Traditional Chinese Medicine Syndrome, The Second Affiliated Hospital of Guangzhou University of Chinese Medicine, Guangzhou, China; ^6^ State Key Laboratory of Dampness Syndrome of Chinese Medicine, The Second Affiliated Hospital of Guangzhou University of Chinese Medicine, Guangzhou, China

**Keywords:** small bowel adenocarcinoma, duodenal adenocarcinoma, ICIS, immunotherapy, chemotherapy

## Abstract

Small bowel adenocarcinoma (SBA), particularly duodenal adenocarcinoma (DA), is a rare gastrointestinal cancer with a dismal prognosis. Data on SBA treatments are limited, and the therapeutic strategy remains uncertain. Currently, chemotherapy is the most used treatment; however, it has a poor median progression-free survival (mPFS) of no more than five months in the second-line setting. We report a case with DA that responded well to the immune checkpoint inhibitor (ICI) tislelizumab plus irinotecan in the second-line treatment. To our knowledge, this is the first report of administering ICIs plus chemotherapy to SBA. Despite the absence of microsatellite instability-high (MSI-H) and high tumor mutational burden (TMB), the patient with *TP53/KRAS* mutation achieved a significantly long PFS of 17 months, and the benefit is still ongoing. The mechanism of this remarkable efficacy might be associated with an increase in tumor immunogenicity after chemotherapy. The current study presents a promising effect of ICIs plus chemotherapy on SBA, affirming the need to investigate the clinical value of this combination in SBA and the underlying mechanism behind it.

## Introduction

Small bowel adenocarcinoma (SBA) is a rare gastrointestinal cancer with a poor prognosis, consisting of 50% duodenal, 30% jejunal, and 20% ileal adenocarcinoma (1). Although there were around 22.7 cases/million in 2004 (2), the incidence of SBA is increasing, with a prevalence in patients over the age of 50 and in men (3). The five-year life expectancy for SBA ranges from 14%–30% (4, 5), whereas the therapeutic options for advanced SBA remain inconclusive. Available data supported chemotherapy as first-line treatment, with a median progression-free survival (mPFS) of six to 11 months (6–8). The optimal mPFS for second-line chemotherapy was only five months (9, 10). Immunotherapy combined with chemotherapy appears to be the cornerstone of treatment for various cancer; however, the efficacy of this combination on SBA has yet to be investigated.

Here, we report the first case of previously treated duodenal adenocarcinoma (DA) with a significant response to second-line tislelizumab in combination with irinotecan. The patient with microsatellite stability (MSS) status, a low tumor mutation burden (TMB), and a *TP53/KRAS* mutation progressed after three months of first-line oxaliplatin-based chemotherapy (XELOX). However, the patient then responded effectively to the combination of immune checkpoint inhibitors (ICIs) and chemotherapy. Our study aims to present the therapeutic potential of ICIs plus chemotherapy in SBA and discuss this combination’s underlying mechanism.

## Current treatments for SBA

### Chemotherapy

There is a dearth of evidence from phase III randomized controlled trials on the SBA treatment. The current therapeutic strategies are mainly derived from phase II studies or retrospective analyses. Oxaliplatin-based regimens (XELOX and FOLFOX) seem to be the most used and effective therapy in the first-line treatment, with an mPFS of six to 11 months and median overall survival (OS) of 15 to 22 months (6–8). In the single agent setting, a retrospective study demonstrated an mPFS of six months and an mOS of 11 months for gemcitabine (11). Triplet chemotherapy regimens, like FAM, CAPIRINOX, and FOLFIRINOX, were also evaluated with a dismal median OS ranging from 8 to 13 months (12, 13). For second-line therapy, an irinotecan-based regimen, FOLFIRI, was recommended with an mPFS of three to five months (9, 10). Taxane-based regimens are other options for second-line treatment, with an mPFS of 3.8 months (14).

### Immunotherapy

The immunotherapy role in SBA is under evaluation. Pembrolizumab is an ideal choice for previously treated patients with MSI-H SBA. Marabelle A’s study included 19 MSI-H patients, and the results showed that pembrolizumab had an ORR of 42.1% and an mPFS of 9.2 months (15). Similar results were observed in studies by Pedersen, K.S (16). and Cardin, D.B (17). However, the mPFS for patients with MSI-L/MSS was only 2.8 months. In Marabelle A’s study (15), only one patient with MSS exhibited a confirmed partial response but correlated with high TMB. These findings suggested that predictive biomarkers may be important for administering immunotherapy in SBA.

### Anti-vascular therapy

A phase II study reported that the mPFS of the XELOX regimen combined with bevacizumab was 8.7 months in first-line treatment (18). Despite the lack of statistical comparison, the mPFS of XELOX plus bevacizumab is comparable to that of XELOX alone, as reported by the same institution (6). However, another retrospective multicenter study reported an mPFS of 15 months in 10 metastatic duodenal and jejunal adenocarcinoma patients treated with bevacizumab plus platinum (19). Notably, among patients treated with bevacizumab-based regimens, the mPFS of six patients with high vascular endothelial growth factor-A (VEGF-A) expression was significantly higher than four patients with low VEGF-A expression, implying that VEGF-A expression might act as a predictor for bevacizumab efficacy.

### Target therapy

It is known that the effect of the anti-epidermal growth factor receptor (EGFR) in colorectal cancer (CRC) depends on the *KRAS* mutation status. Theoretically, approximately 50% of SBA might be treated with anti-EGFR monoclonal antibodies. A case series demonstrated that anti-EGFR might play a role in SBA patients with wild-type *KRAS*. In the study, two *KRAS* wild-type patients had a partial response to cetuximab plus irinotecan, and one showed a complete response (20). In contrast, a phase II study reported unsatisfactory results. Among eight non-mutant *KRAS* SBA patients, panitumumab was administered; however, no clinical responses were observed (21). Moreover, 13 SBA patients with uncertain *KRAS* status were enrolled in a retrospective multicenter investigation. Cetuximab plus chemotherapy was administrated in first- or second-line treatment, and an ORR of 55% was observed (22). Nevertheless, the mPFS of these patients was only 5.5 months, whereas published data showed that SBA patients treated with XELOX or FOLFOX alone could achieve prolonged PFS. More research is required to identify anti-EGFR agents’ efficacy in SBA.

## Case presentation

**Figure 1:**
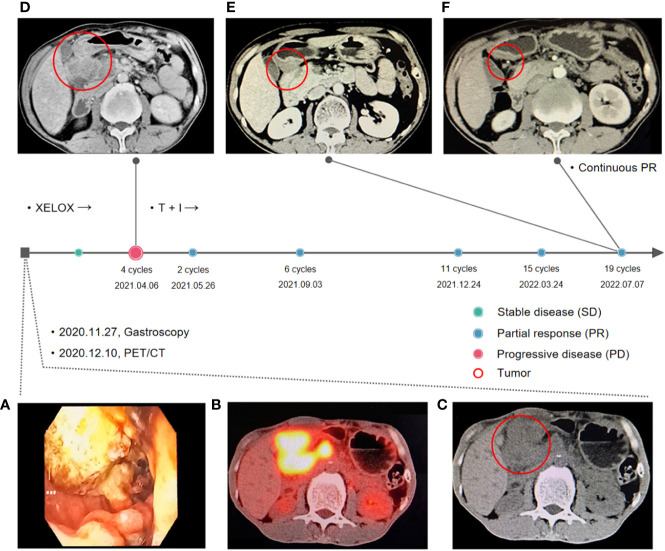
Treatment timeline. **(A)** Gastroscopy report at first visit: A large annular mass was observed in the duodenal bulb, extending to the upper part of the descending part. **(B, C)** Positron emission tomography/computed tomography (PET/CT) before the first-line treatment: a significant uneven thickening of the intestinal wall of duodenal bulb (47 × 40 mm) and multiple lymph node metastases. **(D)** Computed tomography after four sessions of XELOX treatment. The tumor progressed and fused with surrounding lymph nodes after four cycles of XELOX (65 × 63 mm). **(E, F)** Computed tomography after two and 19 sessions of tislelizumab in combination with irinotecan.

The patient was a 50-year-old male. In November 2020, the patient was admitted to a local hospital with a stomachache and a tarry stool. Electronic gastroscopy found a huge ring-shaped mass (**Figure 1A**) in the duodenal bulb, and a poorly differentiated adenocarcinoma was confirmed by biopsy. Subsequently, the patient came to our hospital seeking surgical treatment. Positron emission tomography/computed tomography (PET/CT) indicated a significant uneven thickening of the duodenal bulb’s intestinal wall (47 × 40 mm) and multiple lymph node metastases (**Figures 1B, C**). The margins of the tumor were indistinguishable from surrounding organs (pancreatic head, gallbladder, and liver). The tumor stage was diagnosed as T4N2M0. The gastrointestinal surgeon assessed the tumor as unresectable and referred the patient to our department. We performed a Next-generation sequencing test of circulating tumor DNA to obtain the molecular profile because the patient refused to perform a biopsy again. Results of ctDNA suggested the presence of *TP53 p.S12F KRAS p.G12D* mutations, TMB 2.51 Mut/MB (low), and MSS (Supplementary ctDNA results).

According to published data for advanced SBA, oxaliplatin-based regimens were the most frequently used in first-line treatment with a mPFS of six to 11 months. Therefore, from December 22, 2020, to February 24, 2021, four cycles of the XELOX regimen (oxaliplatin 195 mg day 1, capecitabine 1.5 g bid day 1–14) were administered regularly. After two XELOX cycles, the tumor shrank slightly (35 × 28 mm, **Supplementary Figures S1A, B**), and the response evaluation criteria in solid tumors 1.1 (RECIST 1.1) indicated stable disease. However, at the end of March, the patient appeared with tarry stool again and was admitted to the gastroenterology department at a local hospital for symptomatic treatment. On April 6, 2021, a chest and abdominal contrast-enhanced CT suggested that the tumor progressed and fused with surrounding lymph nodes (65 × 63 mm, **Figure 1D**).

In second-line chemotherapy for SBA, available data suggested that the prognosis was poor regardless of the chemotherapy regimen. On the contrary, patients may benefit from immunotherapy and those sensitive to ICIs could achieve significantly longer survival. Therefore, immunotherapy was considered to be used in the second-line treatment, and chemotherapy was also administered due to the patient having no positive biomarkers associated with immunotherapy. On April 14, 2021, the patient was administered tislelizumab, an immune checkpoint inhibitor, in combination with irinotecan. The giant nodules in the intestinal wall and the lymph nodes shrunk significantly after two sessions of tislelizumab in combination with irinotecan (**Supplementary Figures S1C, D**). After 19 therapy sessions, the giant nodules in the intestinal wall disappeared, and the lymph nodes shrunk significantly (**Figures 1E, F**). Until September 14, 2022, the patient has received 19 cycles of combination therapy of tislelizumab and irinotecan and three cycles of tislelizumab maintenance therapy.

Currently, there is no evidence about chemotherapy combined with ICIs for SBA. Despite the absence of MSS and low TMB, the patient responded well to immunotherapy combined with chemotherapy for 17 months, and the response is still ongoing. No serious adverse events occurred during the treatment. Compared to FOLFIRI regimens with an mPFS of five months in the second-line setting, this combination has achieved great success, which might be mainly attributed to the synergistic effect of immunotherapy and chemotherapy. However, the current study is only one case. The efficacy of the combination of chemotherapy and ICIs in SBA treatment should be further investigated.

## The rationale for combining immunotherapy and chemotherapy in SBA

It was difficult to make a decision on the second-line therapy for the patient. First, the ORR of FOLFIRI in SBA was only 21% and the mPFS was 3.2 months (10), while FOLFIRI may be a better option compared with other regimens. Second, in the first-line therapy, the patient quickly developed resistance to fluorouracil and oxaliplatin, suggesting that it may be inappropriate to use fluorouracil in the second-line treatment. Third, SBA patients with positive biomarkers were sensitive to ICIs and likely to achieve significantly longer survival, but those with MSS/low TMB can hardly benefit from single ICIs (15). The effect of immunotherapy in combination with chemotherapy on SBA has not been reported, although this combination appears to be a cornerstone in the treatment of various cancers. It is well known that regardless of the status of MSS and TMB, ICIs combined with chemotherapy can significantly improve the prognosis of several gastrointestinal malignancies. Chemotherapy not only directly kills tumor cells but also produces a synergistic effect for ICIs by promoting immune recognition and countering immunosuppressive elements (23). On one side, tumor-specific antigens and damage-associated molecular patterns (DAMPs) released by chemotherapy-induced cell death can stimulate the maturation of the antigen-presentation cells and upregulate antigen presentation. In contrast, chemotherapy could modulate suppressive tumor immune microenvironment (TIME) by eliminating immune suppressor cells (regulatory T cells (24) and myeloid-derived suppressor cells (25, 26)) and repolarizing tumor-associated macrophage from M2-like to M1-like phenotype. Therefore, immunotherapy combined with chemotherapy was selected as the patient’s second-line treatment.

## *TP53/KRAS* mutations: Potential immunotherapy biomarkers?

In our case, the patient without MSI-H and high TMB but with co-mutation of *TP53/KRAS* achieved great tumor regression after being treated with irinotecan plus tislelizumab. *TP53* and *KRAS* mutations have been found to exert remarkable effects on TIME in lung cancer, including increasing PD-L1 expression, facilitating T cell infiltration, and augmenting tumor immunogenicity (27). Retrospective analyses suggested *TP53/KRAS* co-mutation might serve as a predictive marker for ICI response in non-small cell lung cancer (27, 28). Thus, we investigated whether *TP53/KRAS* mutations play the same role in gastrointestinal tumors.

**Figure 2:**
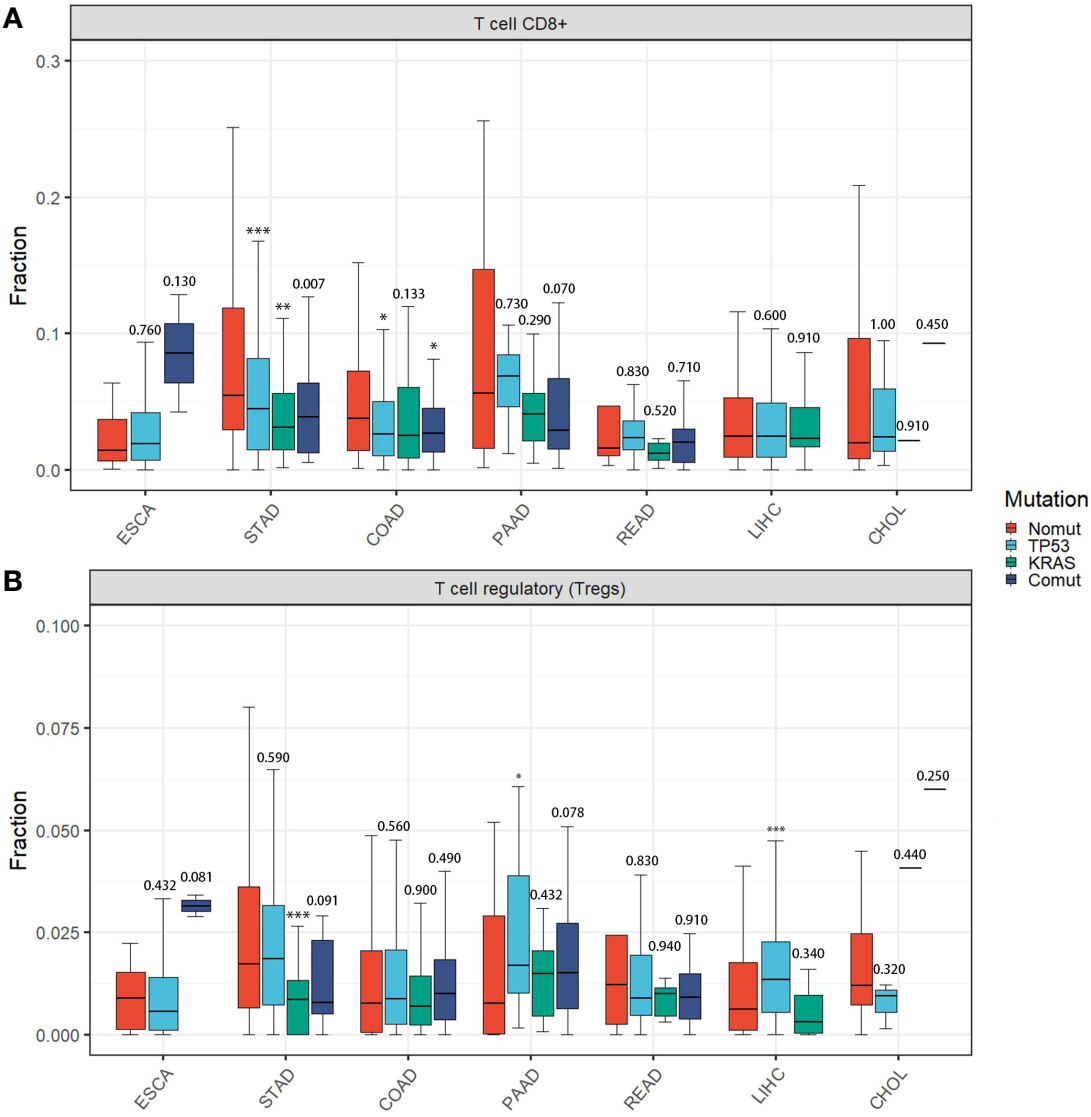
Immune cell infiltration among different TP53/KRAS mutation groups in gastrointestinal tumors. **(A)** T cell CD8+. **(B)** T cell regulatory (Tregs). P-values represented TP53/KRAS mutation groups compared to non-mutant group. (Wilcox test. *, P < 0.05; **, P < 0.01; ***, P < 0.001).

**Figure 3:**
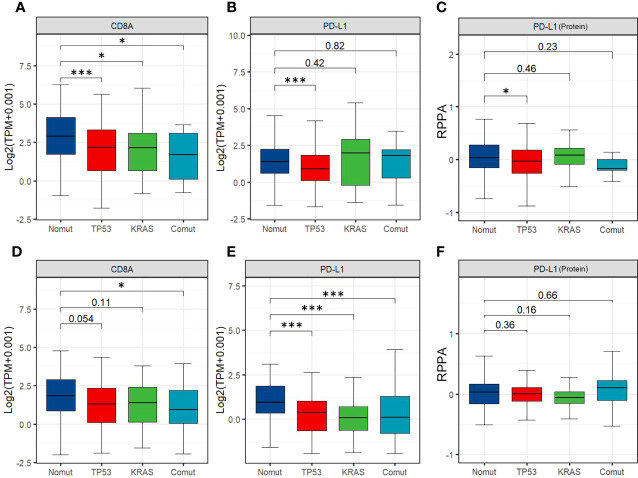
**(A, B)** Expression of CD8A and PD-L1 among STAD'sTP53/ KRAS mutation groups. **(C)** Expression of PD-L1 protein among different TP53/ KRAS mutation groups in STAD. **(D, E)** Expression of CD8A and PD-L1 among different TP53/ KRAS mutation groups in COAD. **(F)** Expression of PD-L1 protein among different TP53/ KRAS mutation groups in COAD. (Wilcox test. *, P<0.05; ***, P<0.001).

Therefore, we assessed the effects of *TP53/KRAS* mutations on TIME, transcriptome, and proteome in gastrointestinal tumors based on The Cancer Genome Atlas (TCGA) database. Seven types of tumors were evaluated, esophageal carcinoma (ESCA), stomach adenocarcinoma (STAD), liver hepatocellular carcinoma (LIHC), cholangiocarcinoma (CHOL), pancreatic adenocarcinoma (PAAD), colon adenocarcinoma (COAD), rectum adenocarcinoma (READ). We found that in STAD and COAD, *TP53/KRAS* mutation groups were associated with the “cold” tumor phenotype (a tumor that is unlikely to benefit from ICIs) (29). In the mutation group, immune cells (CD8+ T cell and regulatory T cell) were less infiltrated (**Figures 2A, B**), and the expression of *CD8A* and *PL-L1* was lower than in the wild-type *TP53/KRAS* group (**Figure 3**). Besides, reduced PD-L1 protein expression was also associated with *TP53* mutation in STAD. Although co-mutation appeared to be associated with a “hot” tumor phenotype in ESCA, the evidence was too weak due to insufficient sample size. Apart from this, no significant difference was detected. Unfortunately, these results did not support our hypothesis that *TP53* and *KRAS* mutations can serve as predictive biomarkers for ICI response in patients with gastrointestinal tumors. From a different perspective, however, this finding suggested that the patient’s significant benefit was more likely to be associated with the combination of immunotherapy and chemotherapy.

## Conclusion

The combination of ICI and chemotherapy should be considered for patients with advanced SBA, particularly duodenal adenocarcinoma.

## Data availability statement

Accession numbers for bioinformatics analysis have been provided in **Supplementary Table 1**. The original contributions presented in the study are included in the article, further inquiries can be directed to the corresponding author.

## Ethics statement

Ethical review and approval was not required for the study on human participants in accordance with the local legislation and institutional requirements. The patients/participants provided their written informed consent to participate in this study. Written informed consent was obtained from the patient for the publication of any potentially identifiable images or data included in this article.

## Author contributions

Conception/Design: XC and H-BZ. Provision of study material or patients: XC and RZ. Collection and/or assembly of data: XC, XQ, and Y-CQ. Data analysis and interpretation: W-ZL, Y-SY and Y-JZ. Manuscript writing: XC and RZ. Final approval of manuscript: L-RL and YL. All authors have read and approved the submitted version of the manuscript.

## Acknowledgments

We thanks to the patient and his family.

## Conflict of interest

The authors declare that the research was conducted in the absence of any commercial or financial relationships that could be construed as a potential conflict of interest.

## Publisher’s note

All claims expressed in this article are solely those of the authors and do not necessarily represent those of their affiliated organizations, or those of the publisher, the editors and the reviewers. Any product that may be evaluated in this article, or claim that may be made by its manufacturer, is not guaranteed or endorsed by the publisher.

## Supplementary material

Supplementary Figure 1 Computed tomography after six **(A, B)**, 11 **(C, D)** and 15 **(E, F)** sessions of tislelizumab in combination with irinotecan.
