# Supplementary figures and images for: Case report: A case of duodenal adenocarcinoma achieving significantly long survival treating with immune checkpoint inhibitors and chemotherapy without positive biomarkers
Source: Front Immunol. 2022 Dec 2;13:1046513. doi: 10.3389/fimmu.2022.1046513 (PMC9755197; doi:10.3389/fimmu.2022.1046513)

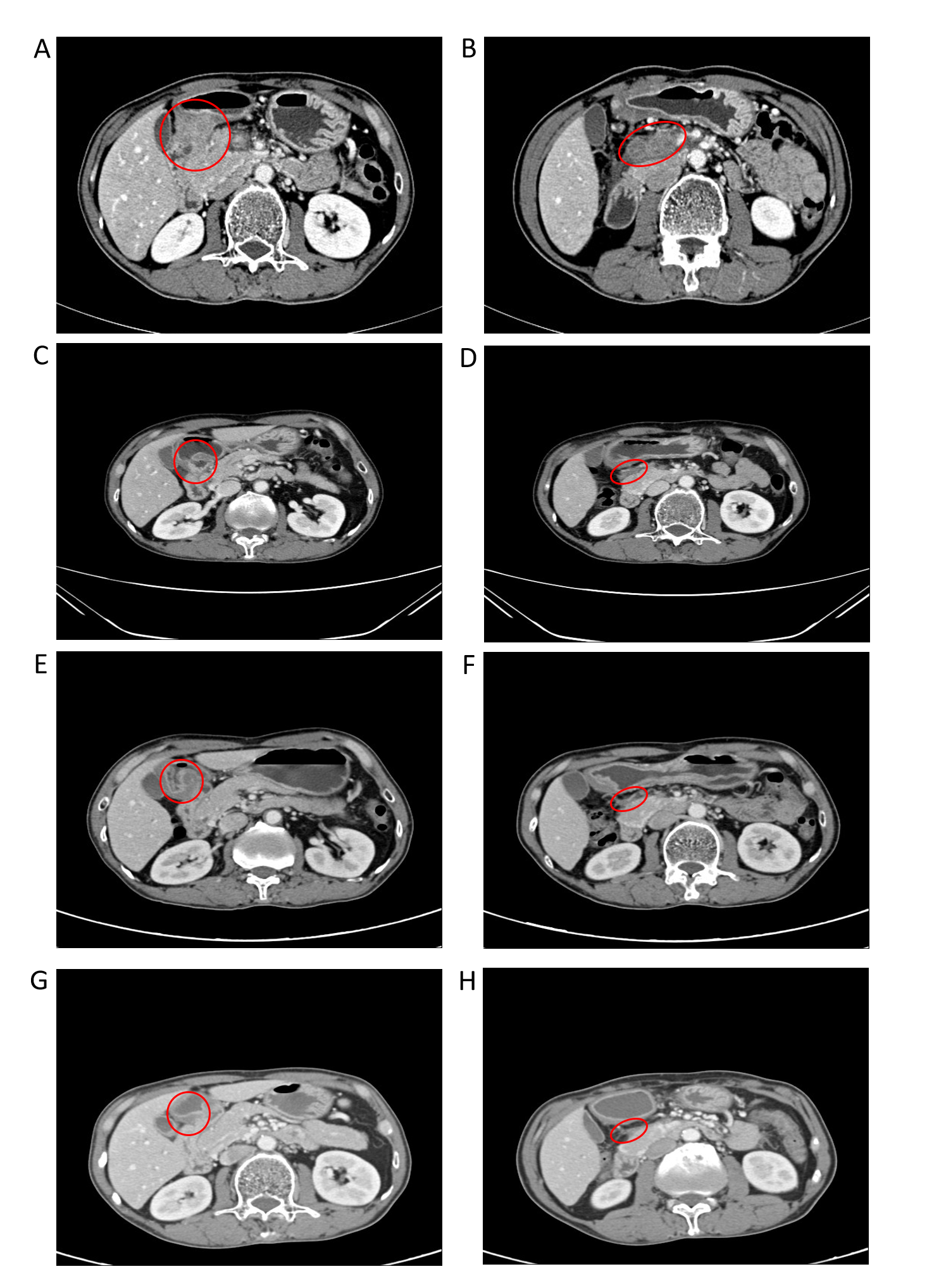

Supplement: Supplementary Figure 1 — Computed tomography after six (A, B), 11 (C, D) and 15 (E, F) sessions of tislelizumab in combination with irinotecan. [file Image_1.tif]
