# Supplementary material for: Case report: A case of duodenal adenocarcinoma achieving significantly long survival treating with immune checkpoint inhibitors and chemotherapy without positive biomarkers
Source: Front Immunol. 2022 Dec 2;13:1046513. doi: 10.3389/fimmu.2022.1046513 (PMC9755197; doi:10.3389/fimmu.2022.1046513)
Supplement: Supplementary file 2 [file DataSheet_1.pdf]

华梵安

肿瘤基因检测报告

Name 姓 名:

Number 编 号:

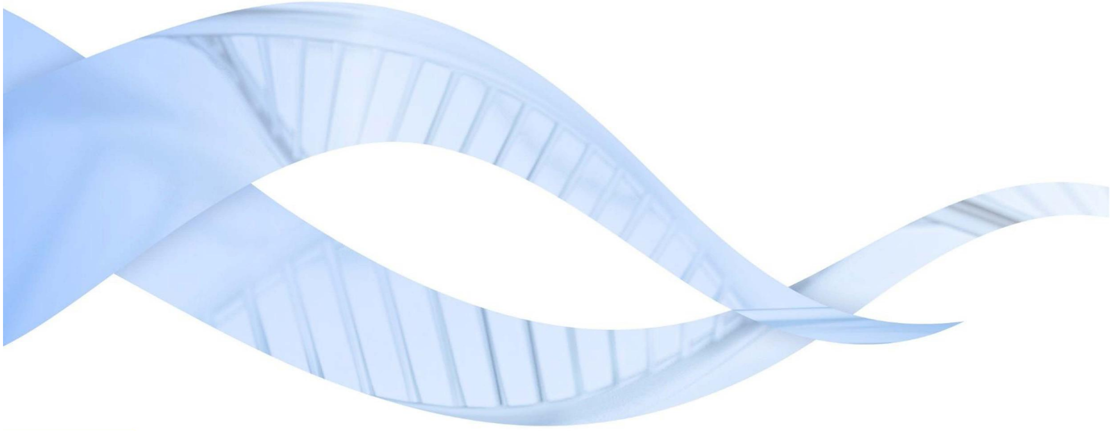

廣東省中醫院
Guangdong Provincial Hospital of Traditional Chinese Medicine

CONTENT 目录

1. 检测概览 Overview 3
1.1 基本信息 Basic information 3
1.2 检测小结 Summary of result 3
1.3 用药提示 Medication Tips 4
1.4 体细胞变异检测结果 Results of somatic cell mutation 4
1.5 胚系变异检测结果 Results of germline variation 5
1.6 临床常规靶向药物相关基因检测结果 Genes associated with targeted theracy 5
1.7 样本质控 Sample Quality Control 6
2.免疫治疗检测结果 7
2.1 肿瘤突变负荷（bTMB） Tumor mutation burden (Blod TMB) 7
2.2 免疫治疗疗效相关基因 Genes associated with immunotherapy 8
3. 临床用药解析 11
3.1 FDA/NMPA 批准用于患者癌种的药物 11
3.2 FDA/NMPA 批准用于其他癌种的药物 12
3.3 可能耐药相关药物 15
3.4 临床试验阶段可能敏感的药物 15
4.基因变异解析 16
4.1 体细胞变异解析 16
4.2 胚系变异解析 18
5. 药物代谢酶 SNP 检测结果 19
6. 可能获益的临床试验 20
7. 产品声明 22
8. 参考文献 24
9.附录 28
9.1 FDA/NMPA/NCCN 提示可能获益的其他药物 28
9.2 检测基因列表 28
9.3 微卫星不稳定性（bMSI） 31

1. 检测概览

1.1 基本信息

姓 名	[redacted]	性 别	[redacted]	年 龄	[redacted]
送检机构	广东省中医院	送检科室	大院胰腺中心	送检医生	吴祥
病理号		住院号	[redacted]		
报告编号		身份证号			
个人其他肿瘤史	无	家族肿瘤史			
用药史					
既往基因检测结果					
Type of sample 样本类型	血浆 Blood	取样部位		样本编号	[redacted]
取样时间		送检时间		到样时间	2020-12-18
肿瘤类型	小肠腺癌	肿瘤分期		报告时间	2020-12-23
临床诊断	Small bowel adenocarcinoma				
病理诊断	腺癌				

1.2 检测小结

Overview 检测结果汇总	Number of Somatic mutation 体细胞变异	11 个	Number of germline mutation 胚系变异	在 69 个肿瘤易感基因中未检测到胚系已知/疑似致病变异 0 /69
	有临床意义变异	4 个	bTMB	2.51 Muts / Mb
	临床意义未明变异	7 个	bMSI	MSS（微卫星稳定）
Targered drug 靶向药物	可能获益 Potentially effective	Binimetinib(D)、阿法替尼 Afatinib+Selumetinib(D)、培唑帕尼(D)、硼替佐米 Bortezomib (D)、索拉非尼+贝伐珠单抗(D) Sorafenib+Bevacizumab		
	可能耐药 Potentially resistant	无 No		
Immunotherapy 免疫药物	可能获益	无 No		
	可能无法获益	无 No		
FDA/NMPA/NCCN 提示其他药物*	可能获益	贝伐珠单抗		
临床试验	可能获益	NCT04214418、NCT04330664、NCT03600883、NCT04418167、NCT03718091 等，详见本报告第 6 章节。		
化疗药物	敏感性可能升高	铂类化合物、环磷酰胺、环磷酰胺+表柔比星联合用药		
	敏感性可能降低	顺铂、卡铂、氟尿嘧啶		

	毒副作用可能降低	顺铂、卡铂、环磷酰胺+表柔比星联合用药、伊立替康、蒽环类、氟尿嘧啶、吉西他滨
	毒副作用可能升高	铂类化合物、依托泊苷+铂类化合物联合用药、伊立替康、蒽环类、氟尿嘧啶

*说明：FDA/NMPA/NCCN 提示其他药物是基于 FDA 或者 NMPA、NCCN 指南对于该癌种用药的提示，并非基于本次基因检测。

1.3 用药提示

Degree of mutation

Results Subregion

检测内容	检测结果		基因亚区	变异等级	FDA/NMPA 已批准用于小肠腺癌的药物		FDA/NMPA 已批准用于其他癌种的药物	临床试验阶段药物
					可能敏感	可能耐药	可能敏感	可能敏感
TP53	p.S127F (c.380C>T)	33.19%	EX5	II 类	-	-	培唑帕尼(D)	-
KRAS	p.G12D (c.35G>A)	19.05%	EX2	II 类	-	-	阿法替尼+Selumetinib (D) 硼替佐米(D) 索拉非尼+贝伐珠单抗(D) Binimetinib(D)	-

This table shows only the relevant mutation for which drug recommendation information is available; see section 1.4 for the results of all somatic mutation.

说明：
1) 本表格仅显示有药物推荐信息的相关变异，所有体细胞变异列表见 1.4 部分；所列药物和临床试验均未按照该患者潜在的预测疗效排序，也未按照其所属肿瘤类型疗效的相关证据可信度排序；
2) 变异等级分类说明、证据等级及相关解释详见本报告第 7 节说明部分。
3) 本报告仅供医生参考，治疗方案由医生决策。

1.4 体细胞变异检测结果

Genes Results Mutation abundance or copy number

基因	检测结果	基因亚区	转录本	突变丰度或拷贝数	变异等级
TP53	p.S127F (c.380C>T)	EX5	NM_000546.5	33.19%	II 类
KRAS	p.G12D (c.35G>A)	EX2	NM_033360.2	19.05%	II 类
CIC	c.3008+1G>T	IVS12	NM_015125.3	32.22%	II 类
CCNE1	拷贝数增加	-	NM_001238.2	5.27	II 类
IGF2R	p.G1844R (c.5530G>A)	EX38	NM_000876.2	21.45%	III 类

MUC6	p.L684_R687del (c.2049_2061delGCTGTCGG [illegible]	EX17	NM_005961.2	15.08%	III 类
CFTR	p.L1077V (c.3229C>G)	EX20	NM_000492.3	8.69%	III 类
MECOM	p.A170V (c.509C>T)	EX6	NM_001105078.3	7.79%	III 类
H3C3	p.R3C (c.7C>T)	EX1E	NM_003531.2	5.32%	III 类
TP63	p.G498C (c.1492G>T)	EX11	NM_003722.4	4.18%	III 类
KMT2C	p.G809R (c.2425G>A)	EX14	NM_170606.2	1.36%	III 类

说明：变异等级分类说明详见第 7 节说明部分。

提示拷贝数变异落在灰区范围，基于 NGS 检测技术局限性，该结果不排除存在假阳性风险；*提示融合断点在当前报告范围内暂时无法注释到编码区。

1.5 胚系变异检测结果

基因	检测结果	基因亚区	转录本	纯合/杂合	遗传方式	临床意义
无	无	无	无	无	无	无

说明：遗传方式：AD表示常染色体显性遗传，AR表示常染色体隐性遗传。常见变异等级分类说明详见第7节说明部分。

1.6 临床常规靶向药物相关基因检测结果 Genes associated with targeted therapy

检测基因	基因突变	检测结果 Results
ALK	重排 Rearrangement	未见突变 No mutation
BCR	重排 Rearrangement	未见突变
BRAF	V600	未见突变
BRCA1	胚系突变 Germline mutation	未见突变
BRCA2	胚系突变	未见突变
EGFR	外显子 18/19/20/21/T790 exon	未见突变
ERBB2	exon 20 外显子 20/拷贝数扩增	未见突变
FGFR2	拷贝数扩增 Copy number amplification	未见突变
FGFR3	拷贝数扩增 Copy number amplification	未见突变
KIT	外显子 9/11 exon 9/11	未见突变
KRAS	密码子 12/13/61/146 codon 12/13..	p.G12D
MET	外显子 14/拷贝数扩增	未见突变
NRAS	密码子 12/13/61	未见突变

NTRK1	重排	未见突变
NTRK2	重排	未见突变
NTRK3	重排	未见突变
PDGFRA	D842V	未见突变
PIK3CA	外显子 10/21（编码外显子 9/20）	未见突变
RET	重排	未见突变
ROS1	重排	未见突变

1.7 样本质控 Quality Control

血浆样本：20P2125001-1			
质控项目		质控结果	质控标准
**DNA 质量评估**	cfDNA 抽提量(ng) cfDNA extraction volume	合格 Qualified	>40ng
**测序质量评估** Sequencing quality assessment	插入片段长度（bp）Length of insert fragment	166bp	[140，210]
	目标区域平均测序深度 Depth of sequencing	2276.83X	>900X
	目标区域覆盖度 Degree of coverage	99.39%	>95%
	碱基测序质量 Q30 比例	Read1 Q30：90.44%<br>Read2 Q30：86.35%	>80%
	样本配对质控	合格	合格

配对样本：20B2125001			
质控项目		质控结果	质控标准
**DNA 质量评估**	DNA 抽提量(ng)	合格	>200ng
**测序质量评估**	插入片段长度（bp）	181bp	[140，210]
	目标区域平均测序深度	510.87X	>300X
	目标区域覆盖度	99.18%	>80%
	碱基测序质量 Q30 比例	Read1 Q30：87.79%<br>Read2 Q30：82.21%	>80%

说明：质控结果分为“合格”和“预警”两个等级，质控结果为“预警”时可能会影响检测的灵敏度和特异性。N/A: not available。

检测者：　　　审核者：　　　报告时间：　2020-12-23

2.免疫治疗检测结果

Results related to immunothetapy

2.1 肿瘤突变负荷（bTMB）

检测内容	检测结果
肿瘤突变负荷（bTMB）	2.51 Muts / Mb

临床意义

血液肿瘤突变负荷（blood-based tumor mutational burden, bTMB）通常指一份肿瘤外周血样本中全外显子测序或靶向测序所检测基因区域每兆碱基中发生的体细胞非同义突变或所有突变的数目。

bTMB 源于肿瘤细胞释放到血液循环中的 DNA(ctDNA)，bTMB 与 tTMB 正相关，可以在一定程度上反映肿瘤组织的 TMB 水平，研究表明 bTMB 与肿瘤组织的 PD-L1 表达无关（PMID：30082870）。

《NCCN 非小细胞肺癌指南》新增组织 TMB（tTMB）作为非小细胞肺癌患者 Nivolumab±Ipilimumab 免疫治疗的生物标志物。目前关于 bTMB 在临床肿瘤治疗中的应用尚未形成共识。

2018 年 Foundation Medicine 发布了首个 bTMB 验证性研究，回顾分析了 Atezolizumab 二线治疗晚期非小细胞肺癌的 OAK（NCT02008227, n=850）和 POPLAR（NCT01903993, n=287）研究。在 POLAR 研究队列中，bTMB（≥10，≥16 和≥20）患者的无进展生存期（PFS）和总生存期(OS)均显著提高。基于 POPLAR 研究队列结果，以 16 SNVs/Mb 为 cutoff 值，OAK 试验人群中 bTMB ≥ 16 SNVs/Mb 患者 Atezolizumab 治疗（n=77）获益优于多西他赛治疗（n=81），无进展生存期(HR: 0.65 (95% CI: 0.47–0.92);P = 0.013)，中位总生存期（mOS）分别为：13.5 个月和 6.8 个月（PMID:30082870）。

另一项基于 Atezolizumab 二线治疗晚期非小细胞肺癌 OAK 试验（NCT02008227, n=318, 训练集）和 POPLAR 试验（NCT01903993, n=106, 验证集）的回顾性研究发现，bTMB（≤ 7 SNVs/Mb)晚期 NSCLC 患者多西他赛疗效优于 Atezolizumab。持续获益（DCB, 定义为总生存期超过 12 个月）患者的 bTMB 显著低于和非持续获益（NDB）组： 5 vs 9 SNVs/Mb; 部分缓解（PR）、疾病稳定（SD）和疾病进展（PD）患者的中位 bTMB 分别为：5 vs 7 vs 10 SNVs/Mb（Annals of Oncology (2019) 30 (suppl_2): ii38-ii68. 10.1093/annonc/mdz063）。

一项 PD-1/PD-L1 单抗单药治疗晚期非小细胞肺癌的前瞻性研究，入组 50 例接受 PD-1/PD-L1 单抗单药治疗的 NSCLC 患者，通过 NCC-GP150（150 基因 Panel）检测 bTMB，研究发现当 bTMB ≥6 定义为 bTMB-H 时，患者免疫治疗的无进展生存（PFS）及客观缓解率（ORR）相比 bTMB-L 患者显著改善（mPFS， NR vs 2.9 m；ORR，39.3% vs 9.1%），且治疗应答组的 bTMB 水平显著高于未应答组。这一差异在 PD-1/PD-L1 作为一线或二线治疗的患者中更为显著（ORR，61.1% vs 6.7%）。无论单因素或多因素分析，bTMB 均是 PFS 和 ORR 的独立预测因素（PMID: 30816954）。

说明:

1) 本产品血浆 TMB（bTMB）计算采用自主研发的 PTAB（Panel-based TMB Analysis process for Blood sample）算法，靶向检测 2.79 Mb 区域，bTMB=突变个数/Mb，其中突变仅指体细胞突变，包括点突变和插入/缺失突变（包含同义突变），去除已报道的驱动突变（与肿瘤治疗、诊断、预后密切相关的突变，包括热点突变、药物靶点突变、癌基因功能激活突变和抑癌基因功能失活突变）。
2) bTMB 与受检者肿瘤类型、诊断分期、样本采集时间、既往治疗方案等因素有关。基于 PTAB 算法的 bTMB 与组织 TMB（tTMB）具有较高的一致性，相关系数 R2=0.71。bTMB 在临床肿瘤治疗中的应用目前尚未形成共识，本报告仅供临床医生参考。
3) 患者的 TMB 值状态评估需基于所有患者的 TMB 分布。但是由于样本突变检出数与测序深度有关，测序深度越高，低频突变检出数越多，因此为尽可能减小测序深度导致的低频突变检出影响，在计算 TMB 时，我们只考虑特定频率阈值以上的变异，以保证同一患者或不同患者间 TMB 值的稳定。
4) 由于本报告的 bTMB 计算去除了可能影响免疫治疗效果的驱动突变，bTMB<0.1 仅表示检测样本在本报告的 bTMB 分析方法的结果，并不等同于检测样本中没有突变。原因可能有：a）样本检测到的突变均为驱动突变，扣除驱动突变后剩余突变数为 0，最终的 bTMB 计算结果为<0.1；b）样本中存在其他突变，这些突变不在本报告的检测范围内。

2.2 免疫治疗疗效相关基因

基因	检测内容	疗效相关预测	检测结果
错配修复缺陷(mismatch repair deficient，dMMR)基因			
MLH1	失活变异	可能获益	未检出
MSH2	失活变异	可能获益	未检出
MSH6	失活变异	可能获益	未检出
PMS2	失活变异	可能获益	未检出
DNA 损伤应答及修复（DDR）相关基因			
ATM	失活变异	可能获益	未检出
ATR	失活变异	可能获益	未检出
BRCA1	失活变异	可能获益	未检出
BRCA2	失活变异	可能获益	未检出
CHEK1	失活变异	可能获益	未检出
FANCA	失活变异	可能获益	未检出
PALB2	失活变异	可能获益	未检出
“超突变(hypermutation)”表型相关基因			
POLE	失活变异	可能获益	未检出
POLD1	失活变异	可能获益	未检出
PBRM1 基因缺陷			
PBRM1	失活变异	可能获益	未检出
PTEN 基因缺陷			
PTEN	失活变异	可能无法获益	未检出
B2M 基因缺陷			
B2M	失活变异	可能无法获益	未检出
JAK1/2 基因缺陷			
JAK1	失活变异	可能无法获益	未检出
JAK2	失活变异	可能无法获益	未检出
EGFR 基因激活			
EGFR	激活变异	可能无法获益	未检出
MDM2/4 扩增			
MDM2	拷贝数增加	可能无法获益	未检出
MDM4	拷贝数增加	可能无法获益	未检出
DNMT3A 基因缺陷			
DNMT3A	失活变异	可能无法获益	未检出
STK11 基因缺陷			
STK11	失活变异	可能无法获益	未检出

说明：本报告仅供医生参考，具体治疗方案由医生决策。
