# Supplementary material for: Case report: A case of duodenal adenocarcinoma achieving significantly long survival treating with immune checkpoint inhibitors and chemotherapy without positive biomarkers
Source: Front Immunol. 2022 Dec 2;13:1046513. doi: 10.3389/fimmu.2022.1046513 (PMC9755197; doi:10.3389/fimmu.2022.1046513)
Supplement: Supplementary file 3 [file DataSheet_2.pdf]

个案报道知情同意书

Patient Consent Form for Case Report

本同意书由作者提供给个案报道的患者本人/监护人/亲属，签字表明知情并同意本论文将在《Frontiers in Immunology》发表患者相关信息。

稿件题目(Titile)：A Case of Duodenal Adenocarcinoma Achieving Significantly Long Survival Treating with Immune Checkpoint Inhibitors and Chemotherapy without Positive Biomarkers

全部作者(Authors)：Xian Chen, Rui Zhou, Yong Li, Xin Qu, Yan-chun Qu, Wen-zhu Li, Yong-song Ye, Li-rong Liu, Yan-juan Zhu.

通讯作者(Corresponding Author)：Hai-bo Zhang

一、我（谢堪冬）特此同意在上述杂志，及与杂志相关的出版媒介上发表与上述主题相关的 我☑ / 我的孩子或受监护人□ / 我的亲属□（请在正确描述后打钩）相关的信息（下称"该信息"）。

二、请在下面两项中适用的一项前面打钩：

☑ 上面文章的作者已经向我详细说明了文章的内容。

☑ 我已经阅读了向该杂志提交的相关资料。

三、我完全明白和理解以下方面内容：

1. 该文章中对我(或患者)的隐私进行了充分保护，与我（或患者）个人身份相关的信息及能辨认出个人身份的照片、图像、文字均不会被披露，我知道并理解。尽管如此，我（或患者）的近亲属及与所接触的经治医护团队仍然可能从文章内容关联到我（或患者）。

2. 该信息将在杂志网站及相关的专业数据库上发布。

3. 该信息的发表面向全世界，该杂志的读者群主要为医护专业人士，用于医学专业交流，但非医生的公众人士，包括记者也有可能获取该信息。

4. 杂志不会将该信息用于广告、包装等商业用途，或节选使用。

5. 我能够在发表前随时撤回我的同意。一旦该信息被交付出版，则不能撤销此同意。

签名：谢堪冬　　与患者关系：本人　　日期：2022.10.8

经办作者签名：周锐　　联系电话：13751880449　　日期：2022.10.8

（以下为空）
